# Mutations in the 3'-untranslated region of *GATA4 *as molecular hotspots for congenital heart disease (CHD)

**DOI:** 10.1186/1471-2350-8-38

**Published:** 2007-06-25

**Authors:** Stella Marie Reamon-Buettner, Si-Hyen Cho, Juergen Borlak

**Affiliations:** 1 Drug Research and Medical Biotechnology, Fraunhofer Institute of Toxicology and Experimental Medicine, Nikolai-Fuchs-Strasse 1, D-30625 Hannover, Germany

## Abstract

**Background:**

The 3'-untranslated region (3'-UTR) of mRNA contains regulatory elements that are essential for the appropriate expression of many genes. These regulatory elements are involved in the control of nuclear transport, polyadenylation status, subcellular targetting as well as rates of translation and degradation of mRNA. Indeed, 3'-UTR mutations have been associated with disease, but frequently this region is not analyzed. To gain insights into congenital heart disease (CHD), we have been analyzing cardiac-specific transcription factor genes, including *GATA4*, which encodes a zinc finger transcription factor. Germline mutations in the coding region of *GATA4 *have been associated with septation defects of the human heart, but mutations are rather rare. Previously, we identified 19 somatically-derived zinc finger mutations in diseased tissues of malformed hearts. We now continued our search in the 609 bp 3'-UTR region of *GATA4 *to explore further molecular avenues leading to CHD.

**Methods:**

By direct sequencing, we analyzed the 3'-UTR of *GATA4 *in DNA isolated from 68 formalin-fixed explanted hearts with complex cardiac malformations encompassing ventricular, atrial, and atrioventricular septal defects. We also analyzed blood samples of 12 patients with CHD and 100 unrelated healthy individuals.

**Results:**

We identified germline and somatic mutations in the 3'-UTR of *GATA4*. In the malformed hearts, we found nine frequently occurring sequence alterations and six dbSNPs in the 3'-UTR region of *GATA4*. Seven of these mutations are predicted to affect RNA folding. We also found further five nonsynonymous mutations in exons 6 and 7 of *GATA4*. Except for the dbSNPs, analysis of tissue distal to the septation defect failed to detect sequence variations in the same donor, thus suggesting somatic origin and mosaicism of mutations. In a family, we observed c.+119A > T in the 3'-UTR associated with ASD type II.

**Conclusion:**

Our results suggest that somatic *GATA4 *mutations in the 3'-UTR may provide an additional molecular rationale for CHD.

## Background

GATA4 (MIM# 600576) is a transcription factor which is characterized by a highly conserved binding domain of two zinc fingers. It is expressed in the heart and is essential for mammalian cardiac development (see reviews [1-3]). In mice, germline ablation of the gene encoding Gata4 results in abnormal ventral folding of the embryo, failure to form a single ventral tube, and lethality [4,5]. Besides heart development, Gata4 is involved in the formation of multiple organs, such as intestine, liver, pancreas and swim bladder in zebrafish [6] as well as gastric epithelial development in mouse through interaction with Fog co-factors [7].

In humans, four *GATA4 *gene mutations have been identified in families with congenital heart defects (CHD) notably, atrial septal defects [8-11]. One mutation (p.Gly296Ser) affects a highly conserved amino acid after the C-terminal zinc finger, and disrupts physical interaction with TBX5 [8], a transcription factor described to be defective in Holt-Oram syndrome [12]. But *GATA4 *germline mutations are rather rare. Two studies analyzed 16 families each, but only 2/16 families with multiple affected members carried *GATA4 *mutations [10,11]. In 42 patients with non-syndromic atrioventricular septal defects (familial as well as sporadic), no *GATA4 *mutations have been detected [13].

It is of considerable importance that genetic analysis of blood samples may not reveal somatically-occurring sequence alterations in the diseased cardiac tissues, but these mutations may provide a molecular rationale for pathogenesis [14,15]. We recently reported evidence for somatically-derived *NKX2-5 *and *TBX5 *mutations as a novel mechanism for septation defects of the human heart [16-18], and used the same heart collection of malformed hearts to investigate *GATA4 *gene mutations, notably those affecting the coding of amino acids for zinc finger functions of the protein [19]. To identify further genetic alterations associated with CHD, we continued our search in the 609 bp of 3'-untranslated region (3'-UTR) of *GATA4*.

The 3'-UTR of *GATA4 *is relatively long and likely contains regulatory elements essential for the regulation and transport of the mRNA transcript [20]. Indeed, evidence is accumulating that the 3'-UTR of mRNA is involved in the control of nuclear transport, polyadenylation status, subcellular targetting as well as rates of translation and degradation of mRNA; thus sequence alterations in this region may lead to disease (see review [21,22]). Recently, a single nucleotide deletion in the 3'-UTR of high mobility group A1 gene (*HMGA1) *was identified in two type 2 diabetes patients exhibiting deficiency of the protein [23]. The mutation decreased *HMGA1 *mRNA half life in the lymphoblast cells of the patients, and further deletion of the 3'-UTR of *HMGA1 *resulted in the decrease of reporter assay expression. To the best of our knowledge, the 3'-UTR of *GATA4 *has not been investigated particularly as regards its role in post-transcriptional gene expression. In this paper, we report the detection of frequent *GATA4 *sequence alterations in the 3'-UTR in the diseased tissues of malformed hearts, some of which are predicted to alter RNA secondary structure. As a result of aberrant RNA folding, these mutations may lead to CHD by affecting mRNA localization and translation of protein. We found further five nonsynonymous mutations in exons 6 and 7 of *GATA4*.

## Results

**Figure 1:**
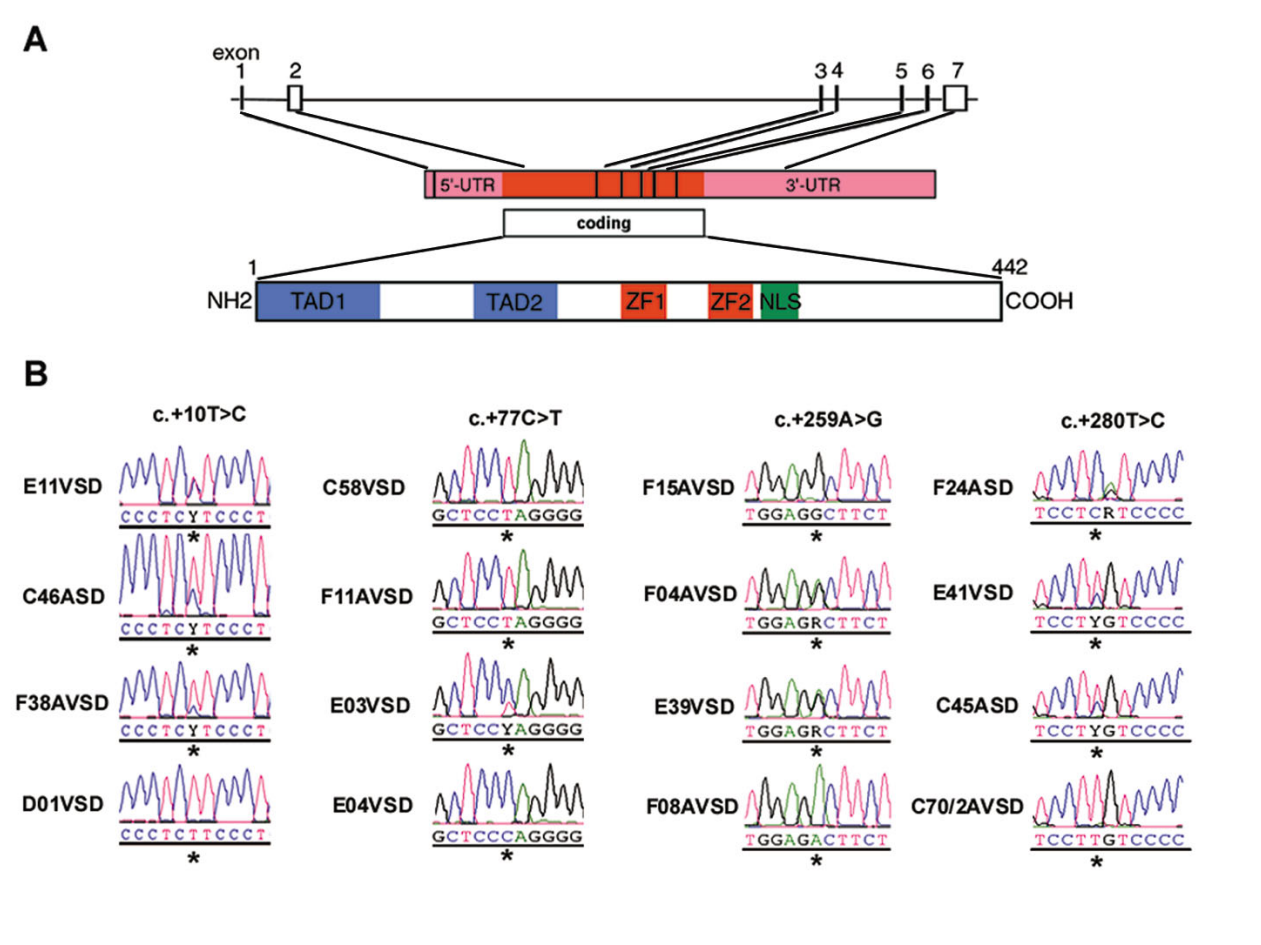
Genetic analysis of *GATA4*. **(A) **Schematic representation of GATA4 (genomic, mRNA and protein level). Reference sequence for *GATA4 *was based on NM_002052 and the intron-exon boundaries on [42]. **(B) **Examples of 3'-UTR mutations and genotypes obtained after direct sequencing of fragments amplified from diseased cardiac tissues of patients affected by CHD. Additional sequence alterations in the 3'- UTR of *GATA4 *were detected, e.g. F24ASD (c.+281G>A).

*GATA4 *is located on chromosome 8p23.1-p22, consists of seven exons and codes for a 442- amino-acid protein (see Fig. 1A). We investigated the genomic DNA for sequence variations in the entire coding region and untranslated regions (UTR) of this gene. In the case of formalin-fixed material, exons 3, 4, 6 and 7 of malformed hearts of patients with complex CHD could be amplified. We reported earlier the detection of 23 nonsynonymous mutations in exons 3 and 4, notably mutations affecting the zinc fingers of GATA4 [19]. Here we report further five nonsynonymous mutations in exons 6 and 7, which affect highly conserved amino acids 361, 377, 430, 432 and 442 in the C-terminal region of GATA4 (Table 2). Whether these sequence alterations result in aberrant protein expression requires additional studies. Previously, it has been shown that a frameshift mutation past amino acid 359 severely altered the encoded GATA4 protein and was associated with ASD [8]. Nonetheless, mutations p.Leu432Ser and p.Ala442Thr identified by us would affect polarity of the protein, due to replacement of non-polar, hydrophophic amino acids with polar, hydrophilic ones. In addition, p.Met361Val likely results in aberrant RNA folding, as determined by a method described below.

**Table 2:** Summary of *GATA4 *sequence variations in diseased cardiac tissues of patients with CHD

Nucleotide change NM_002052	Coding region	Amino acid change	Location	Positive hearts (n = 29 VSD)	Positive hearts (n = 16 ASD)	Positive hearts (n= 23 AVSD)	Total	PCR-RFLP assay
NONSYNONYMOUS**								
1599	c.1081A>G	p.Met361Val	exon 6	1			1	
1648	c.1130G>A	p.Ser377Asn	exon 6	1			1	
1806	c.1288C>G	p.Leu430Val	exon 7		1	1	2	
1813	c.1295T>C	p.Leu432Ser	exon 7		1		1	
1842	c.1324G>A	p.Ala442Thr	exon 7	1			1	
**3'UNTRANSLATED REGION**								
1857	c.+10T>C		exon 7	1	2	4	7	*Earl*I
1891	c.+44T>A		exon 7	1		3	4	
1924	c.+77C>T		exon 7	2*		3*	5	*Sty*l
2065	c.+218C>T		exon 7	1		3	4	*Earl*I
2106	c.+259A>G		exon 7	1	1	2*	4	
2127	c.+280T>C		exon 7	1	3*	1	5	
2289	c.+442A>G		exon 7	1	1	1	3	
2309	c.+462T>C		exon 7	1	4	1	6	
2326	c.+479A>G		exon 7	1*	1	1	3	*Nar*I
2201	c.+354A>C		exon 7, dbSNPrs867858	27AA:2AC	12AA:4AC	19AA:3AC:1CC	58AA:9AC:1CC	*Msp*A1I
2273	c.+426C>T		exon 7, dbSNPrs1062219	1CC:8CT:20TT	2CT: 13TT	1CC: 4CT:18TT	2CC:14CT:51TT	
2364	c.+517T>C		exon 7, dbSNPrs884662	9TT:16CT:4CC	4TT:11CT	4TT:15CT:4CC	17TT:42CT:8CC	
2379	c.+532T>C		exon 7, dbSNPrs904018	1TT:9CT:19CC	9CT:6CC	1TT:3CT:19CC	2TT:21CT:44CC	
2410	c.+563C>G		exon 7, dbSNPrs12825	15CC:8CG:6GG	2CC:12CG	15CC:6CG:2GG	32CC:26CG:8GG	
2434	c.+587A>G		exon 7, dbSNPrs804291	1AG:28GG	13GG	23GG	1AG:64GG	

* homozygous genotypes observed

** nonsynonymous mutations in exons 3 and 4 reported earlier (Reamon-Buettner and Borlak, J Med Genet 42:e32)

**Figure 2:**
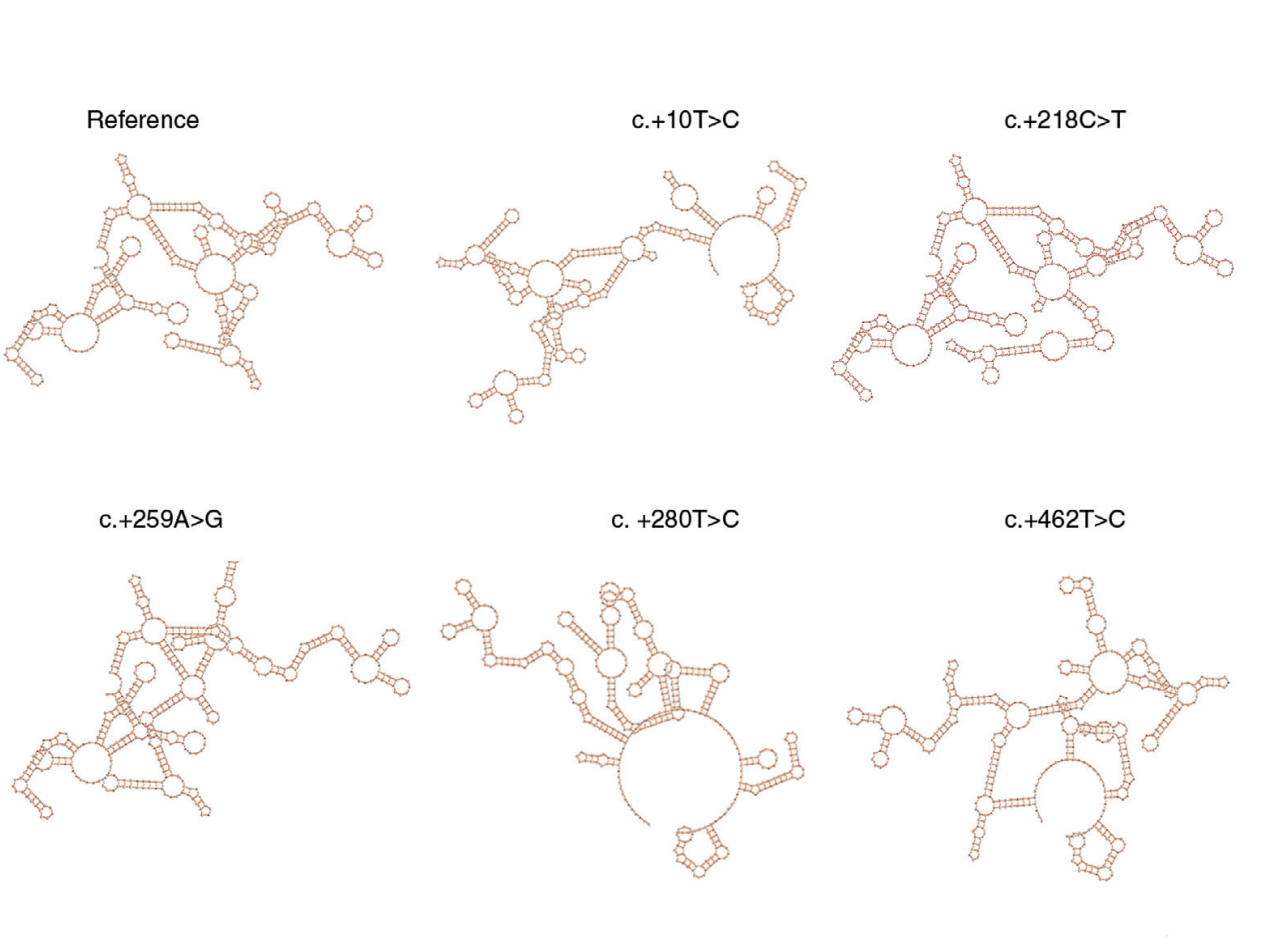
Effect of mutations in the 3'-UTR of *GATA4 *on mRNA folding. Using the program GeneQuest (Lasergene 6.0), the effect of sequence alterations on RNA folding was predicted using the first c.+500 nt in the 3'-UTR of *GATA4*. GeneQuest uses the Vienna RNA folding procedure, taken from Zuker's optimal RNA folding algorithm [43], to fold the sense strand of selected DNA regions as RNA. Compared to the reference sequence, 7/9 mutations would result in faulty RNA folding, on which five patterns here are shown. Same pattern was observed for c.+10 T > C and c.+44T > A.

Notably, exon 7 of *GATA4 *consists of 1,708 bp, the majority (1,525 bp) being untranslated. From our collection of formalin-fixed hearts, we could amplify the first four fragments of exon 7 (see Fig. 1A and Table 1 for the primer sequences designated GTx7-1 to GTx7-4). These primer sequences cover a part of the coding region c.1221 to c.1329 (nt 1739–1847, NM_002052) and 3'-UTR from c.+1 to c.+609 (nt 1848–2456, NM_002052). We found 9 sequence alterations, occurring in 3 to 7 patients, and 6 dbSNPs in the analyzed 3'-UTR region of *GATA4 *(Table 2, Table 3). On the basis of sequence electropherograms and/or PCR-RFLP assays, both heterozygous and homozygous genotypes were observed (Fig. 1B). Using the program GeneQuest (Lasergene 6.0), we determined the effect of the nine sequence alterations on RNA folding. GeneQuest uses the Vienna RNA folding procedure, taken from Zuker's optimal RNA folding algorithm, to fold the sense strand of selected DNA regions as RNA. We investigated the first 500 bp of the 3'-UTR, simply because the sequence alterations identified by us are located within this segment. Furthermore, there is suggestion that the whole 3-'UTR is not necessary for localization, but that localization signals lie within the regions of < 100–200 nt [25]. Compared to the reference sequence, 7/9 of these would result in faulty RNA folding (Fig. 2). While c.+77C > T and c.+479A > G would likely not lead to faulty RNA folding, the same pattern was observed for c.+10T > C and c.+44T > A. In addition, we searched the 3'-UTR of *GATA4 *for highly conserved motifs that may be involved in post-transcriptional regulation, (see [26]). None of the conserved motifs reported so far have been detected, but the highly conserved motif (AATAAA) associated with polyadenylation signals were located at positions c.+678–683 and c.+1502–1507.

**Table 1:** Primer sequences and PCR conditions used for analysis of *GATA4 *mutations

*GATA4 *exons	Exon size (bp)	Primer	Primer length	Primer sequence (5'-3')	Melting temperature	PCR product length (bp)
exon 1	61	GT4x1F	20	cttgcacgtgactcccacag	65°C	250
		GT4x1R	20	aagcaaaggcggagaagctc		
exon 2	1073	GT4x2-1F	21	tctctttctgtcgttcctctt	65°C	600
		GT4x2-1R	20	gcacgtagactggcgaggac		
		GT4x2-2F	21	ggaccatgtatcagagcttgg	65°C	641
		GT4x2-2R	20	gccctcgcgctcctactcac		
exon 3	167	GT4x3F	24	agtcagagtgaggaagagcaagag	65°C	541
		GT4x3R	21	cagtttctgtgtgccgaagag		
exon 4	126	GT4x4F	20	ccagccctgcctcccgttag	65°C	294
		GT4x4R	23	gaggactgagagatgggcatcag		
exon 5	88	GT4x5F	22	agtagccatcacatcacacagg	65°C	504
		GT4x5R	19	aaagctcccaacacgttcc		
exon 6	149	GT4x6F	20	gtttgtccctgccgctgatt	65°C	247
		GT4x6R	20	gcagtcggcctccccacaaa		
exon 7	1708	GTx7-1F	19	acaaggctatgcgtctccc	60°C	289
		GTx7-1R	20	ctgagaaaatccaacacccg		
		GT4x7-2F	20	gcgtaatcttccctcttccc	65°C	291
		GT4x7-2R	19	gggacaaggacatcttggg		
		GTx7-3F	20	gtcgacaatctggttagggg	60°C	281
		GTx7-3R	20	gtacatggcaaacagatgcc		
		GTx7-4F	20	gaggatctgagaacaagcgg	60°C	270
		GTx7-4R	20	cagctgcattttgatgaggc		
		GTx7-5F	22	aaattgtggggtgtgacataca	60 °C	577
		GTx7-5R	22	gttgcagaatctctggcttttt		
		GTx7-6F	22	ctgtctgtctgctcctcctagc	65°C	586
		GTx7-6R	22	acctcccagtgaagaccactaa		

**Table 3:** Spectrum of *GATA4 *mutations (nonsynonymous and 3'-UTR) in diseased tissues of malformed hearts

Patient	Age	Total defects*	Detected mutations		
			
			Coding (ns)***	3'-UTR	Total
**VSDs**					
C06VSD**	1h	3	F211L, Y244C, R260Q, C292R		4
C10VSD	stillborn	6	C292R		1
C19VSD	3 mo	4	C292R		1
C58VSD	18 d	5	C292R	c.+77C>T	2
D01VSD	aborted	3	C292R		1
D03VSD	2 mo	4	C292R		1
E03VSD**	6 mo	2		c.+77C>T	1
E04VSD**	5 wk	2	C292R		1
E08VSD	53 yr	1	C292R		1
E10VSD	6 mo	4	G214S		1
E11VSD	newborn	1		c.+10T>C	1
E13VSD	16 yr	1	N239S, C292R		2
E15VSD	8 mo	2	N239D		1
E16VSD	3 mo	3	**M361V**		1
E20VSD	2 d	3			0
E22VSD	4 mo	3	F208L, C292R		2
E23VSD**	22 d	3	L261P, C292R		2
E26VSD	6 mo	3	C292R, **S377N**	c.+479A>G	3
E27VSD	5 mo	2	M223T, R229S, C292R	c.+462T>C	4
E28VSD	5 mo	2		c.+44T>A	1
E29VSD	8 wk	3	C292R		1
E31VSD	5 mo	2			0
E33VSD	14 yr	1	**A442T**		1
E34VSD	3 mo	2			0
E35VSD	4.5 mo	2	C292R		1
E39VSD	9 mo	3	C292R	c.+218C>T, c.+259A>G	3
E40VSD	15 mo	1	C292R		1
E41VSD	23 yr	2	C292R	c.+280T>C	2
E49VSD	newborn	3	C292R	c.+442A>G	2
**ASDs**					
C09ASD	26 yr	2			0
C39ASD	25 yr	4	C292R		1
C45ASD	6 d	7	C292R, **L432S**	c.+280T>C, c.+462T>C	4
C46ASD	34 yr	3	C292R	c.+10T>C, c.+259A>G, c.+462T>C	4
C64ASD	11 mo	4	C292R		1
C75ASD	2 mo	6	C292R		1
D30ASD	11 yr	3	**L430V**		1
D33ASD	8 yr	2	N248S, L261P	c.+462T>C, c.+479A>G	4
F17ASD	4 1/2 h	1		c.+280T>C	1
F19ASD	8 yr	4		c.+462T>C	1
F20ASD	10 yr	1			0
F24ASD	6 yr	3		c.+280T>C	1
27ASD	7 yr	2	C292R	c.+442A>G	2
F29ASD	14 yr	8	I255T, A294V	c.+10T>C	3
F30ASD	1 d	2			0
F32ASD	38 yr	1	R229S		1
**AVSDs**					
C43AVSD	10 mo	7	H302R	c.+10T>C	2
C70/2AVSD**	8 mo	6	G234S, R252P, C292R		3
C71AVSD**	4 d	5		c.+10T>C, c.+442A>G	2
E05AVSD	16 wk	6		c.+10T>C	1
E36AVSD**	3.5 mo	4			0
E43AVSD	4.5 mo	4	R283H		1
F00AVSD**	6 wk	5		c.+44T>A, c.+218C>T	2
F02AVSD	10 mo	7	N248S	c.+77C>T	2
F03AVSD**	1 mo	4		c.+44T>A	1
F04AVSD**	11 wk	5	C292R	c.+218C>T, c.+259A>G	3
F05AVSD**	10 wk	4	F211L, R229S, Y244C, N285K		4
F08AVSD**	5 mo	5	P226fs		1
F11AVSD**	7 mo	6	C292R	c.+77C>T	2
F12AVSD	6 mo	5	R266X		1
F13AVSD**	25 mo	5	C292R		1
F14AVSD**	1 mo	5	T277I, C292R		2
F14aAVSD**	5 wk	5		c.+462T>C	1
F15AVSD	5 d	7		c.+218C>T, c.+259A>G	2
F18AVSD**	7 mo	5			0
F28AVSD	8 yr	3	N273S, **L430V**		2
F31AVSD**	8 d	6		c.+479A>G	1
F38AVSD	3 mo	4	C292R	c.+10T>C, c.+77C>T, c.+280T>C	4
F40AVSD	10 mo	3		c.+44T>A	1

* Heart malformations described earlier in Reamon-Buettner et al. 2004 Am J Pathol 2004, 164:2117–2125.

** Down syndrome

*** nonsynonymous *GATA4 *mutations in exons 3 and 4 reported earlier in Reamon-Buettner and Borlak 2005, J Med Genet 42:e32; new mutations detected in exons 6 and 7 are in bold

To compare healthy vs. diseased tissues from each patient, we selected specifically those who were positive for mutations in the diseased tissue. For instance, in the diseased tissues, we found 17/68 patients without nucleotide changes in analyzed region of the 3'-UTR except for the dbSNPs. Thus for this comparison, we analyzed 21 patients for GTx7-1 (21 × 2 × 289 bp = 12,138 nucleotides); 25 patients for GTx7-3 (25 × 2 × 281 bp = 14,050 nucleotides) and 24 patients for GTx7-4 (24 × 2 × 270 bp = 12,960 nucleotides). Except for the dbSNPs rs867858, rs1062219, rs884662, rs904018, rs12825 (see Table 2), none of the mutations were detected in the unaffected tissues. This result suggests somatic origin and mosaicism of mutations.

In the formalin-fixed malformed hearts, we observed six dbSNPs which were all located within 234 nucleotides in exon 7 (Table 2). We could calculate the allele frequencies for five loci (Table 2) and found the dbSNPs to be in Hardy-Weinberg equilibrium. We cloned three fragments from hearts heterozygous for rs1062219 (c.+426C > T), rs884662 (c.+517T > C) and rs904018 (c.+532T > C). Surprisingly, we found three haplotypes after sequencing of at least four clones in each heart. In all three hearts, we found one type containing all reference alleles [c.+426C; c.+517T; c.+532T] and another type with all variant alleles [c.+426T; c.+517C; c.+532C]. Additional types found were [c.+426T; c.+517T; c.+532T] with one variant allele, and [c.+426C; c.+517C; c.+532C] with two variant alleles.

**Figure 3:**
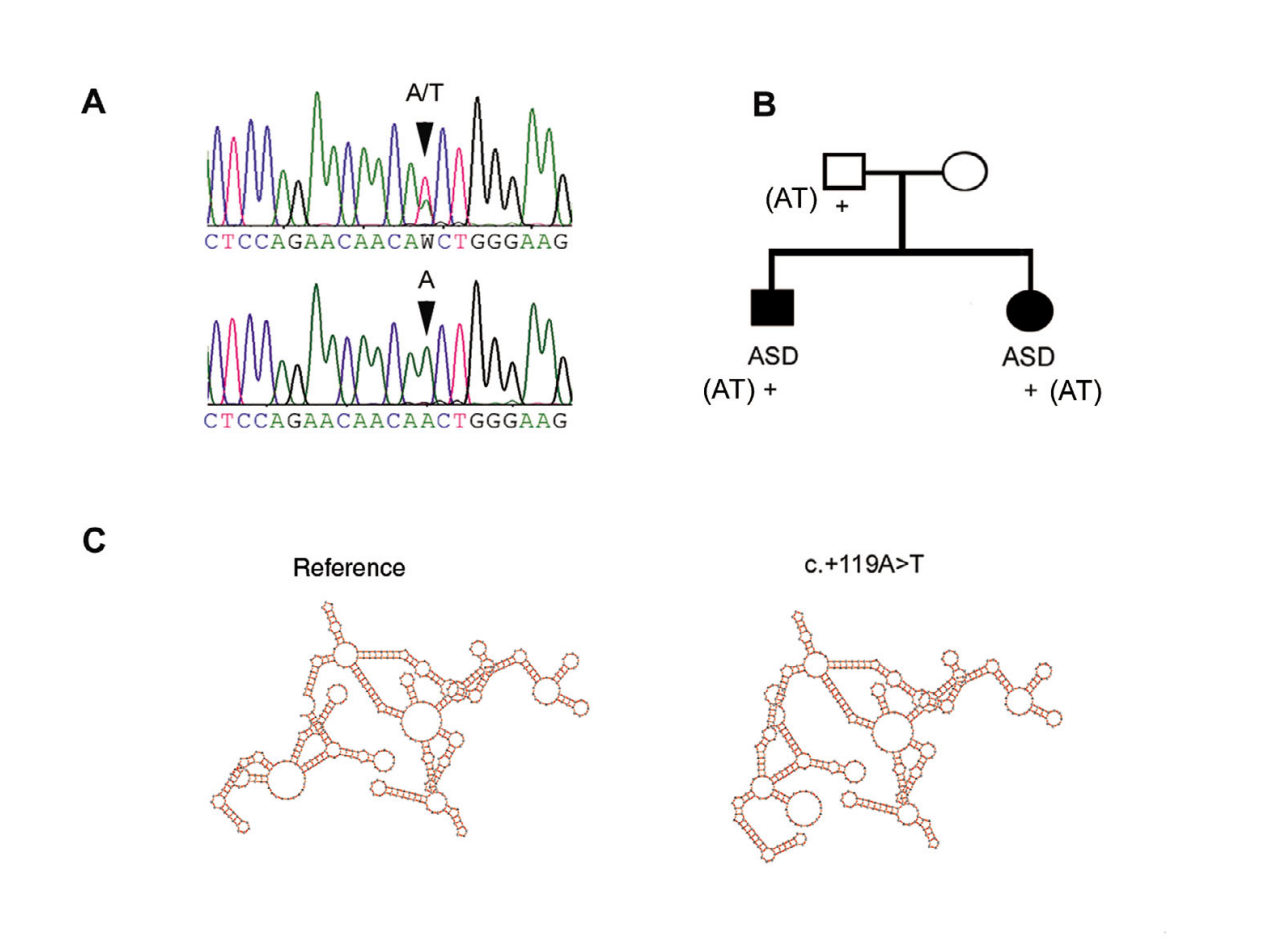
Detection of 3'-UTR mutations in blood samples of patients with CHD. (A) sequence electropherograms for c.+119A>T, showing heterozygous (AT) and homozygous (AA) genotypes; (B) in a family of four in which the two children had ASD; the father and the children had the heterozygous genotype AT (+), the mother was homozygous for the reference allele AA; (C) Compared to the reference sequence and on the basis of Vienna RNA folding procedure, c.+119A>T is predicted to affect RNA folding.

We further analyzed the entire gene in blood samples of 12 patients with CHD (see Fig. 1A). Mostly dbSNPs were detected (Table 4); nonetheless we observed two sequence variations in the 3'-UTR (c.+119A > T and c.+1260G > A) and two intronic (c.-518-25C > T and c.-458+5A > G) which have not yet been reported as dbSNPs. The 3'-UTR alterations and c.-518-25C>T were detected in a family of four, in which the two children were affected by ASD, but the parents had no history of CHD. For c.+119A>T, the mother was homozygous for the reference allele AA, while the father and the children had the heterozygous genotype AT (Fig. 3A&B). This sequence variation would affect RNA folding (Fig. 3C). As control, we analyzed blood samples from 100 unrelated Caucasian healthy individuals. No sequence variations were found, except for a synonymous change in exon 3 (c.699G>A, p.233Thr), an intronic (c.783+16G>A) and dbSNPs (Table 4).

**Table 4:** Summary of *GATA4 *sequence variations in blood samples

Nucleotide change NM_002052	Coding region	Amino acid change	Location	NCBI dbSNP number	Blood samples CHD	Blood samples normal
**NONSYNONYMOUS**						
1647	c.1129A>G	p.Ser377Gly	exon 6	rs3729856	10AA:2AG	
**SYNONYMOUS**						
1217	c.699G>A	p.Thr233	exon 3			1 (GA)
**3'-UTR**						
1967	c.+119A>T		exon 7		2 (AT)	
3107	c.+1260G>A		exon 7		1 (GA)	
2201	c.+354A>C		exon 7	rs867858	3AA:9AC	53AA:36AC:11CC
2273	c.+426C>T		exon 7	rs1062219	1CC:8CT:3TT	39CC:38CT:23TT
2364	c.+517T>C		exon 7	rs884662	4TT:5CT:3CC	43TT:30CT:27CC
2379	c.+532T>C		exon 7	rs904018	1TT:5CT: 6CC	30TT: 30CT:40CC
2410	c.+563C>G		exon 7	rs12825	8CC:3CG:1GG	69CC:11CG:20GG
2434	c.+587A>G		exon 7	rs804291	12GG	100 GG
3005	c.+1158C>T		exon 7	rs11785481	7CC:5CT	
3103	c.+1256A>T		exon 7	rs12458	3AA:9AT	
3202	c.+1355G>A		exon 7	rs1062270	11GG:1AG	
3368	c.+1521G>C		exon 7	rs3293358	5CC:6CG:1GG	
**INTRONIC**						
	c.-518-25C>T		intron		2 (1CT:1TT)	
	c.-458+5A>G		intron		1 (AG)	
	c.783+16G>A		intron			1 (GA)
	c.614-61G>C		intron	rs10503425	8GG:3CG:1CC	27AA:12AC:3CC
	c.997+56A>C		intron	rs804280	3AA:7AC:2CC	

## Discussion

Recent studies characterize further the importance of Gata4 in cardiac development. Indeed, small changes in the level of Gata4 protein expression can dramatically influence cardiac morphogenesis and embryonic survival [27]. Moreover, myocardial expression of *Gata4 *is required for proliferation of cardiomyocytes, formation of the endocardial cushions, development of the right ventricle, and septation of the outflow tract [28]. To gain further insights into the role of *GATA4 *mutations in CHD in humans, we have analyzed the Leipzig collection of malformed hearts for sequence alterations of this gene. We reported previously the identification of 23 nonsynonymous mutations, 19 of which are located within the highly conserved zinc fingers and basic regions of GATA4 [19]. Notably, we identified a frequent mutation (p.Cys292Arg) in VSDs, which affects one of the four zinc coordinating cysteines in the C-finger and is predicted to disrupt the secondary structure of the protein. Functional characterization of site-directed mutated residues in the C-finger, showed that disruption of the zinc coordinating cysteines, would abolish DNA binding, Gata4-Nkx2-5 interactions and synergy [29,30].

To identify genetic alterations associated with CHD, we continued our search for mutations in the 3'-UTR region of *GATA4*. In the diseased tissues of malformed hearts, we found frequent alterations in the 3'-UTR of *GATA4*, in which 7/9 would affect RNA folding. Additional sequence alterations in the 3'- UTR of *GATA4 *were detected, but these were mostly isolated cases and therefore not reported here. Further, we identified a germline mutation (c.+119A>T) in which two affected family members were positive. Similarly, it would alter RNA folding. Since the unaffected father carried c.+119A>T, we cannot discount the possibilities of a low penetrance mutation or no causal association at all. Whether the 3'-UTR mutations reported here contributed to cardiac malformations by affecting the secondary structure of mRNA or through other post-transcriptional regulation associated with 3'-UTR, requires further functional analysis. Nonetheless, it has been shown that two mutations in the 3'-UTR of rat *Mti *(metallothionein-1) that are predicted to alter the secondary structure of this region also impaired localization of the mRNA [25].

There is, however, accumulating evidence on the important role of 3'-UTR in post-transcriptional regulation. For instance, efficient termination and stability of mRNA are dependent on a properly configured 3'-UTR [31]. Localization of mRNAs is also due to specific sites or signals within the 3'-UTR [25,32]. Recently, a systematic search discovered 106 highly conserved motifs in human 3'-UTRs that may be involved in post-transcriptional regulation, including mRNA stability and degradation [26]. Many of these are associated with microRNAs, which are non-coding RNAs of 21–22 nucleotides and are complementary to the 3'-UTR motifs that mediate post-transcriptional regulation [33]. Similary, 53 sequence motifs have been catalogued in 3'-UTR of yeast mRNAs, including motifs corresponding to known RNA-binding protein sites and motifs associated with subcellular localization [34]. Furthermore, 83 disease-associated variants in the 3'-UTR of human protein-coding genes have been systematically analyzed, and that functionality of these variants correlated highly to predicted secondary structural changes [35].

Many studies are suggestive for Gata4 to network with other transcription factors to regulate cardiac gene expression (see reviews [1-3]). These protein-protein interactions are mediated through the proteins' binding domains and for Gata4, the zinc fingers are involved. For instance, N-terminal zinc finger interacts with Fog2, while the C-terminal zinc finger contacts Nkx2-5, Nf-At3, Mef2c and Hand2. Notably, the C-finger of Gata4 interacts with the third alpha helix (homeodomain) of Nkx2-5, to enable transcriptional synergy of regulated genes. Site-directed mutagenesis of zinc coordinating cysteines in the C-finger leads to abrogation of Gata4-Nkx2-5 interaction and loss of synergy [29,30]. We reported previously the identification of somatically-derived GATA4 zinc finger mutations in the same malformed hearts, including a frequent mutation (p.Cys292Arg) in VSDs, which affects one of the four zinc coordinating cysteines and is predicted to disrupt the secondary structure of the protein [19]. However, aside from p.Cys292Arg and unlike *NKX2-5 *[16,17] common nonsynonymous mutations are rare in the coding regions of *GATA4*. Thus, the further detection of mutations in the 3'-UTR of *GATA4 *in the same malformed hearts with zinc finger mutations suggests a possible further mechanisms of disease, in which the zinc fingers are not involved. Indeed, as shown in Table 3, in which *GATA4 *nonsynonymous and 3'-UTR mutations identified in hearts with septation defects are summarized, there were 14 with 3'-UTR mutations only. These consist of 3 in VSDs, 3 in ASDs, and 8 in AVSDs. From these 14 hearts, 12 carried 3'-UTR mutations that are predicted to result in aberrant RNA folding. As can be surmised from Table 3, it appears that 3'-UTR mutations are more frequent in ASDs and AVSDs than in VSDs. In VSDs, 23 out of 29 had mutations in the coding region, most of which were p.Cys292Arg and only 9 out of 29, carried 3'-UTR mutations. It is also of considerable interest that sporadic cases of CHD from Lebanese population were analyzed for mutations affecting the zinc fingers and basic region of GATA4 [36]. A mutation (p.Glu216Asp) in the N-terminal zinc finger was detected in 2/26 patients with Tetralogy of Fallot (TOF), and this mutation resulted in reduced transcriptional activity without affecting GATA4's ability to bind DNA, nor its interaction with ZFPM2 (FOG2). However, none of the other 94 patients with various CHD phenotypes carried any mutations.

To help elucidate genetic alterations in affected tissues of malformed hearts, we have investigated in the past a panel of cardiac transcription factor genes from the same 68 malformed hearts [16-19,37]. Direct sequencing revealed mutations in diseased tissues, which were basically absent in matched normal heart samples. Common occurring mutations were identified, especially in the binding domains of transcription factors, which could affect DNA-protein or protein-protein interactions leading to CHD. While certain transcription factor genes (*NKX2-5*, *GATA4*) exhibited a high rate of mutations, others were not or rarely affected (*HEY2, MEF2C*). Results of these studies enabled us to put forward a hypothesis of somatic mutations as a novel molecular cause of CHD. Furthermore, we found malformed hearts containing combination of mutations in the binding domains of several transcription factors, As example, we identified in two patients with AVSD combined mutations in the binding domains of HEY2, NKX2-5, TBX5 and GATA4 [37]. We also identified 21 patients with combined mutations in the GATA4 zinc fingers and the homeodomain of NKX2-5 (unpublished results).

Our malformed hearts were stored in formalin more than 40 years ago and there is speculation about artifacts arising from this procedure [38-40]. To ensure reliability of our findings, we investigated sequence variations in 10 formalin-fixed, but healthy hearts which were collected at the same time as the malformed hearts. Essentially, these hearts were free of *GATA4 *mutations, therefore providing convincing evidence for reliability of the assay. In addition, our identification of six dbSNPs within the 3'-UTR demonstrates faithful preservation of DNA and reliability in using valuable retrospective material with the obvious advantage of studying clearly defined pathology, as opposed to using surrogate tissue material distal to organ defect. Five dbSNPs were highly polymorphic (see Table 2) and were found to be in Hardy-Weinberg equilibrium. This was confirmed with our control population of healthy individuals. But cloning of fragments amplified from diseased heart tissues of patients who were 'heterozygous' for three dbSNPs detected three haplotypes instead of the expected two, which may be due to duplication of *GATA4 *in analyzed tissues.

This result is similar to our previous observations on different cardiac transcription factors conducted on the same set of malformed hearts [16-19,37]. After cloning amplified fragments with several closely-spaced 'heterozygous' mutations as marker loci within a single gene, we observed several haplotypes in individual hearts, instead of two haplotypes as expected of a diploid genome. (Note, we used the term haplotype to define the set of alleles within an investigated gene locus). The cause of the observed multiple haplotypes is unknown, but may be explained by a mixed population of cardiomyocytes carrying different mutations or de novo chromosomal rearrangements and gene duplications in the heart tissues of patients affected by CHD. Our results for NKX2-5 by using a yeast-based assay to determine function suggest that different haplotypes can lead to different cardiac disease phenotypes [41]. Furthermore, the presence of combined mutant alleles may alter/modify pattern of mRNA folding. We found, for instance, patients who were either positive for c.+218C>T or c.+259 A>G or both. Two patients were heterozygous for both mutations, while one patient was homozygous for both variant alleles. Singly c.+218C>T or c.+259 A>G would lead to misfolding (see Fig. 2), but if combined only the pattern observed for c.+259 A>G would result. Moreover, different haplotypes due to combinations of closely-spaced polymorphisms in the 3'-UTR of genes can result in different mRNA stabilities in transient expression assays [35]. Nonetheless, as the malformed hearts were conserved in formalin, possibilities exist that the observed multiple haplotypes could be PCR errors resulting from fragmented DNA. We believe the contrary, however, as specific haplotypes were detected in several malformed hearts after cloning, but were absent in matched unaffected heart tissue (unpublished results).

To further support DNA assay reliability on the formalin-fixed material, we examined in the course of our work with the Leipzig heart collection, additional genes which have been associated with cardiac malformations. We searched for sequence variations affecting the binding domains of MEF2C and HEY2. In the case of *MEF2C *gene, no nonsynonymous mutations have been detected in a total of 68 patients with complex CHD (unpublished results). A similar finding was observed with the *HEY2 *gene, where only three nonsynonymous mutations were found in 2 of 52 patients [37]. Our work on *TBX5 *in the same heart collection likewise supports reliability of using the material [18]. Mutations in this T-box transcription factor has been associated with Holt-Oram syndrome (HOS), a disorder characterized by heart and upper limb deformities [12]. We amplified 200, 307, 276, 203 and 515 bp containing exons 3, 4, 5, 7 and 8, respectively in the same heart collection. The sequences encoding the T-box are located in exons 3, 4 and 5. We found a total of nine nonsynonymous mutations distributed in few patients with ASDs and AVSDs. None of the 29 VSDs carried any nonsynonymous mutations, only two synonymous mutations were found in two patients. To assess further method reliability, we investigated in the same patient cohort amplified fragments from other genes, with or without prior implication to heart development (e.g. *CFC1, HMGN4 *and *BRCC2)*, in the same heart collection, as well as in formalin-fixed normal mouse heart *s (e.g. Hand1, Nkx2-5 *and *Gata4) *but did not find sequence alterations [37].

## Conclusion

We identified hotspots associated with cardiac defects in the 3'-UTR region. Our results suggest that somatic *GATA4 *mutations in the 3'-UTR may provide an additional molecular rationale for CHD

## Methods

Materials, genomic DNA isolation, and mutation detection have been described previously [16]. Briefly, we analyzed 68 formalin-fixed hearts of Caucasians with complex cardiac malformations, notably n = 29 with ventricular (VSD), n = 16 with atrial (ASD), and n= 23 with atrioventricular (AVSD) septal defects. The explanted hearts, which were collected between 1954–1982, were obtained from the Institute of Anatomy, University of Leipzig, Germany. We also analyzed blood samples of 12 Caucasian patients with CHD, and blood samples of 100 unrelated healthy Caucasian individuals. In blood samples, defects of patients with CHD included VSD, ASD, hypoplastic left heart syndrome (HLHS), transposition of the great arteries (TGA), sub-pulmonary stenosis (SPS) and heterotaxy. Except for two patients who came from the same family, 10/12 patients were unrelated individuals. In the formalin-fixed malformed hearts, diseased tissues in the vicinity of the septal defects were analyzed. Matched healthy heart tissues were likewise investigated for sequence alterations. Materials used in this study were obtained in accordance to an approved protocol. JB has obtained approval to conduct genetic studies involving human materials from the Medical School Hannover. The formalin-fixed hearts were collected more than 40 years ago and cadavers were donated voluntarily by patients' relatives.

The primers and PCR conditions used in investigating sequence variations of *GATA4 *are given in Table 1. PCR-amplified fragments were sequenced directly using BigDyeTerminator v3.1 Kit (Applied Biosystems, Darmstadt, Germany) and Applied Biosystems 3100 Genetic Analyzer. Sequences were analyzed using SeqScape 2.0 (Applied Biosystems) or DNASTAR Lasergene 6.0 (Madison, Wisconsin USA). The numbering of mutations within the coding region of *GATA4 *starts with nucleotide A of the first codon ATG, while that in the untranslated region was based on NM_002052. Nucleotide changes in the untranslated and intronic regions were numbered according to suggested nomenclature [24]. Sequence variations were verified by independent PCR, double-strand sequencing, PCR-RFLP, or cloning of heterozygous genotypes followed by subsequent sequencing of clones, allowing the identification of variant alleles. Unless reported as NCBI dbSNPs, we refer to nucleotide changes as sequence variations or mutations interchangeably, which simply means deviations from the reference *GATA4 *sequence (NM_002052), and disease-causing or not.

## Abbreviations

CHD Congenital heart disease

ASD Atrial septal defect

VSD Ventricular septal defect

AVSD Atrioventricular septal defect

UTR Untranslated region

## Competing interests

The author(s) declare that they have no competing interests.

## Authors' contributions

SMRB participated in the conceptual design of the study, was responsible for the lab work, carried out analysis and interpretation of the data, and drafted the manuscript. SHC participated in the screening, confirmation, and analysis of mutations as part of her M.D. project. JB was in-charge of the conceptual design of the study, participated in the analysis and interpretation of the data, and in the final writing of the manuscript. All authors have read and approved the final manuscript.

## Pre-publication history

The pre-publication history for this paper can be accessed here:


